# Somatic Embryogenesis in Selected Conifer Trees *Pinus nigra* Arn. and *Abies* Hybrids

**DOI:** 10.3389/fpls.2019.00013

**Published:** 2019-01-29

**Authors:** Terézia Salaj, Katarína Klubicová, Radoslava Matusova, Ján Salaj

**Affiliations:** Institute of Plant Genetics and Biotechnology, Plant Science and Biodiversity Center, Slovak Academy of Sciences, Nitra, Slovakia

**Keywords:** *Abies* hybrids, cryopreservation, embryogenic cultures, genetic transformation, *Pinus nigra*

## Abstract

Somatic embryogenesis was achieved in the conifers *Pinus nigra* Arn. and in the hybrids *Abies alba* ×*A. cephalonica* and *Abies alba* ×*A. numidica*. For initiation of embryogenic tissue in *P. nigra*, immature zygotic embryos enclosed in megagametophytes were used. The initiated embryogenic cultures were maintained and proliferated on solid culture medium DCR supplemented with 9 μM 2,4-D and 2.2 μM BA. Microscopic investigations revealed the presence of bipolar early somatic embryos in proliferating tissue. Suspension cultures have also been established by resuspending the embryogenic tissue in liquid culture medium. Experimentation with abscisic acid concentration resulted in successful somatic embryo maturation. Besides abscisic acid, the carbohydrate content or higher concentration of gelling agent in maturation medium were also important requirements for somatic embryo maturation. Germination of cotyledonary somatic embryos occurred on hormone-free medium and terminated in somatic seedlings regeneration. The regenerated somatic seedlings were transferred to soil and were capable of successful development. For initiation of embryogenic tissue in *Abies* hybrids juvenile explants as immature or mature zygotic embryos as well as cotyledons were used and 4.4 μM BA as sole plant growth regulator was sufficient. Medium of the same composition was also suitable for their long-term maintenance. Maturation of somatic embryos was achieved on solid DCR medium supplemented with 38 μM abscisic acid, polyethylene glycol (0, 5, 7.5, and 10% PEG-4000) and different carbohydrates such as maltose, sucrose and glucose (each 3%). PEG-4000 stimulated somatic embryo development depending on the carbohydrate source used. Cotyledonary somatic embryos germinated after desiccation treatment and the regenerated somatic seedlings were transferred to soil. Cryopreservation of embryogenic tissue could be an alternative method for long-term maintenance. For cryopreservation the slow-freezing method was used with success. Tissue regeneration in the post thaw period was relatively high and the regenerated tissue produced mature somatic embryos and subsequent plantlets. The embryogenic tissue was also used in experiments focused on genetic transformation either by biolistic (*P. nigra*) or *Agrobacterium*-mediated (*Abies* hybrids) methods. A proteomic study was performed to gain a deeper insight into the early stages of *P. nigra* somatic embryogenesis.

## Introduction

*Pinus nigra* Arn. is a medium to large conifer that is native of the area from central to southeastern Europe to western Asia. The altitudinal distribution of *P. nigra* is between 250 and 1800 m (Mirov, 1967). Due to its biological and economic importance, the species was the subject of studies focusing on its genetic diversity (Rubio-Moraga et al., 2012), paleobiogeography (Roiron et al., 2013), adaptability to soil conditions (Arsova, 1999) and fire history (Touchan et al., 2012).

Planting of *P. nigra* in Central Europe started in the early twentieth century with the aim of stopping soil erosion on slopes originally covered by grazed rock grassland (Anton et al., 2008). The species is one of the most important introduced trees in Slovakia, and adapted well to local ecological conditions in the forest type group *Querceto*-*Fagetum* (Tokár, 1985). It was also demonstrated that *P. nigra* trees are suitable bioindicators of environmental pollution in Slovakia (Ostrolucká et al., 1995; Mičieta and Murín, 1998).

Several attempts have been made to propagate the species using *in vitro* techniques (micropropagation). Micropropagation was based on adventitious bud initiation on mature zygotic embryos (Kolevska-Pletikapič et al., 1983; Salajová, 1993) or axillary bud development on shoot tips (Salajová, 1992). Plantlets have also been developed but the rooting efficiency was relatively low.

Another native species, silver fir (*Abies alba* Mill), is also a very important conifer in Central Europe. However, starting in the second half of the last century, the populations of silver fir in Slovakia have declined due to the effect of higher pollution in the environment. This low adaptability of *Abies* species to rapidly changing environmental conditions may be a consequence of a high degree of genetical uniformity of the Central European populations of silver fir. So, the planting of more resistant individuals, obtained either by artificial hybridization or by *in vitro* approaches, has been recommended.

An extensive hybridization program, started in 1975, developed several intra- and interspecific *Pinus* and *Abies* hybrids (Kormut’ák, 1985; Kormut’ák et al., 1992, 2012).

The hybrid nature of the obtained seedlings was confirmed by restriction analysis (Kormutak et al., 2013). Attempts were made at effective multiplication of obtained hybrid seeds, mostly by micropropagation *via* adventitious bud initiation and subsequent plantlet regeneration (Vooková et al., 1989; Vooková and Gajdošová, 1992). Adventitious buds developed on cotyledons of 7-day-old seedlings using BAP (5 mg.l^-1^) combined with NAA (0.01 mg.l^-1^). Shoot elongation was stimulated by application of spermidine but the rooting of elongated shoots was very low. Owing to the low efficiency of the mentioned micropropagation methods, protocols for somatic embryogenesis were developed in *P. nigra* and several *Abies* hybrids.

Somatic embryogenesis of conifer species, first described for Norway spruce, proved to be promising method for *in vitro* propagation (Chalupa, 1985; Hakman et al., 1985). Since the first description of somatic embryogenesis in Norway spruce, the process has been initiated in large numbers of conifer species, including several genera, and the results have been reviewed (Tautorus et al., 1991; Attree and Fowke, 1993; Salaj et al., 2015; Klimaszewska et al., 2016).

This review focuses on the results obtained for *P. nigra* and *Abies* hybrids somatic embryogenesis by our research group, including germplasm conservation through cryopreservation and genetic transformation.

## Somatic Embryogenesis in *Pinus nigra* Arn.

### Initiation of Embryogenic Tissue in *Pinus nigra*

Somatic embryogenesis in *Pinus* species was mostly initiated from juvenile explants as immature zygotic embryos. Due to the small size of zygotic embryo in this developmental stage, megagametophytes with developing zygotic embryos as explants were cultured on nutrient medium containing plant growth regulators (auxins and cytokinins). This approach was successfully used for *P. sylvestris* (Aronen et al., 2009), *P. radiata* (Hargreaves et al., 2009; Montalbán et al., 2012), *P. patula* (Jones and van Staden, 1995), *P. halepensis* (Montalbán et al., 2013), *P. pinea* (Carneros et al., 2009), *P. monticola* (Percy et al., 2000). The disadvantage of using immature zygotic embryos is that these are available only in a short period of the year, so it would be desirable to extend the “initiation window” to more advanced developmental stages of explants. In *P. radiata* somatic embryogenesis was initiated from differentiated tissues of post-cotyledonary zygotic embryos (Find et al., 2014). For *P. contorta* successful somatic embryogenesis initiation was reported from shoot buds collected from adult trees (Park et al., 2010).

The embryogenic tissue is initiated after several days to several weeks of explant cultivation. After initiation the tissue is maintained and proliferated by repeated subcultures on a fresh medium every 2–3 weeks. The media used for proliferation are usually the same as for initiation. Occasionally, for continual proliferation of tissues medium, modification was necessary. In *P. elliottii* Engelm. reduction of plant growth regulators concentrations to one-tenth supported sustained tissue growth (Newton et al., 1995). Longer cultivation of tissues by repetitive transfers on fresh media may result in loss of maturation ability, particularly in *Pinus* species (Klimaszewska et al., 2009). This phenomenon could be alleviated using maltose based medium devoid of plant growth regulators, or using weekly subcultures (Breton et al., 2005).

**FIGURE 1:**
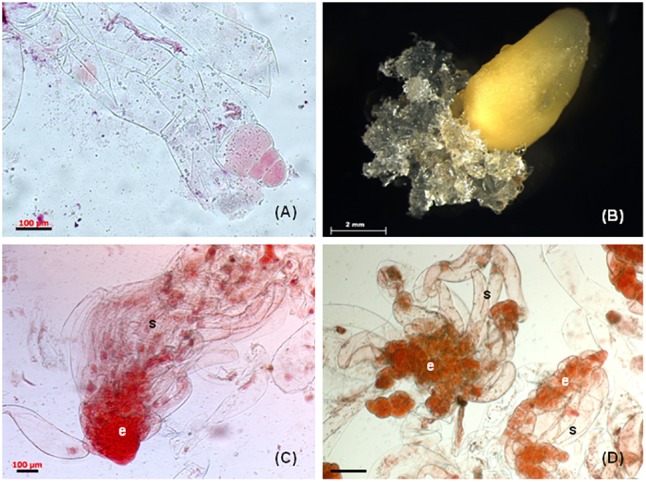
Somatic embryogenesis in *Pinus nigra*. **(A)** zygotic embryo at responsive stage enclosed in megagametophyte explant, **(B)** extrusion of mucilaginous embryogenic tissue from micropylar end of megagametophyte, **(C)** bipolar early somatic embryo stained with acetocarmine, the embryonal cells (e) are tightly packed and the long vacuolised suspensor cells (s) are arranged into bundle, **(D)** early somatic embryo, the embryonal cells (e) are loosely connected and the suspensor cell (s) are without organization into bundle. Scale bars: **(A,C)** = 100 μm, **(B)** = 2 mm, **(D)** = 200 μm.

Several attempts were made to initiate somatic embryogenesis in *P. nigra* from different types of juvenile explants and only immature zygotic embryos enclosed in megagametophytes produced embryogenic tissue (Salajová et al., 1995, 1999). Immature precotyledonary zygotic embryos isolated from female gametophytes dried out shortly after being placed on the semi-solid culture media, resulting in no initiation (Salajová et al., 1995). Cotyledonary zygotic embryos isolated from mature seeds produced only friable non-embryogenic callus (Salajová et al., 1999). To determine the developmental stage of zygotic embryos in megagametophytes, immature seeds were opened and the embryo carefully excised and stained with 2% acetocarmine. This approach confirmed that the zygotic embryos used at the sampling time were in the precotyledonary developmental stage (Figure 1A). The white mucilaginous tissue extruded from the micropylar end of the megagametophyte explants (Figure 1B) and proliferated rapidly. The tissue was separated from the primary explant and cultured individually as a cell line once it reached approximately 5 mm in size.

One of the most important factors affecting the initiation of embryogenic tissue from conifer species is the developmental stage of the zygotic embryo. Observations in many *Pinus* species have suggested that the zygotic embryos have the potential to produce embryogenic tissue at a very early developmental stage (Lara-Chavez et al., 2011; Montalbán et al., 2012; Alvarez et al., 2013). Our earlier experiments have confirmed this phenomenon in *P. nigra* and the observed data indicated the importance of cone collection time (i.e., zygotic embryo developmental stage) for embryogenic tissue initiation (Salajová et al., 1995). As the zygotic embryos in megagametophytes developed, the initiation frequencies dropped from 8.03 to 1.17 or 0%, regardless of the culture medium used. Based on these results, subsequent experiments used precotyledonary embryos enclosed in megagametophytes as the preferred explants for somatic embryo initiation in *P. nigra* (Salajová et al., 1999; Salajová and Salaj, 2005; Salaj et al., 2014). Immature zygotic embryos were also used as explants for *P. nigra* J.F. Arnold subsp. *nigra* var. *caramanica* (Loundon) Businsky (Özkurt et al., 2008) and mature zygotic embryos for *P. nigra* Arn. ssp. *salzmannii* (Radojevic et al., 1999).

Another factor that plays a role in embryogenic tissue initiation in conifer species is the mineral composition of the basal medium. In *P. nigra*, media differing in inorganic salt composition such as DCR (Gupta and Durzan, 1985) and LV (Litvay et al., 1981), with full or half concentration of macro- and microelements such as MLV (Klimaszewska et al., 2001) or QP (Quoirin and Lepoivre, 1977), respectively, were tested during two consecutive years. In regard to initiation frequencies, media DCR and MLV were superior over LV or QP. Initiation frequencies of 5.2–10.4% (DCR) and 7.3–8.8% (MLV) were obtained (Table 1). Very low initiation frequencies were obtained using LV or QP (Salaj et al., 2014). The effect of low concentrations of PGRs suggested by Klimaszewska et al. (2001) was also tested for *P. nigra*. In our experiments, concentration of 2,4-D in the culture medium was lowered from 9 to 2.25 μM and the experiments were performed over several consecutive years (5 seasons). The beneficial effect of lowered 2,4-D concentration was significant only for the 1st year and no significant differences were observed in the next period (Salaj et al., 2014).

**Table 1:** Initiation of somatic embryogenesis from immature zygotic embryos of *Pinus nigra* using different culture media.

Medium	Years			
	2007		2008	
	IF	Number of initiated cell lines	IF	Number of initiated cell lines
DCR	5.2 (1.84)^a,c,d^	5.0	9.6 (0.7)^e^	13.0
DCR-R	10.4 (1.4)^a,c^	14.0	10.3 (0.93)^e^	14.0
LV	2.1 (1.4)^c^	2.0	0.0^e,f^	0.0
LV-R	0.0^d^	0.0	0.0^e,f^	0.0
MLV	8.3 (2.81)^b^	8.0	7.4 (2.72)^f^	10.0
MLV-R	7.3 (2.86)^b^	7.0	8.8 (1.79)^f^	12.0
QP	0.0^b,c^	0.0	0.74 (0.73)^e,f^	1.0
QP-R	0.0^b,c^	0.0	0.0^e,f^	0.0


*Initiation frequencies (IF) are expressed as % of explants producing embryogenic tissues per total number of cultured explants. (With permission from Salaj et al., 2014: Dendrobiology71, 119–128).For each medium 96 explants were cultured in 2007 and 136 explants in 2008 (altogether 1856).Standard errors of mean are in parenthesis.^*a*^Statistically significant at (P 0.05) within the given year, ^*b,c,d*^statistically significant at p 0.01 within the given year, ^*e,f*^statistically significant at p 0.01 within the given year.Media DCR, LV, MLV, QP were with 9 μM 2,4-D and 2.2 μM BA.Media DCR-R, LV-R, MLV-R, QP-R were with 2.25 μM 2,4-D and 2.2 μM BA*.

Some researchers have tried to improve the somatic embryogenesis process by modifying the basal medium based on the mineral element composition of megagametophytes (seed tissue). Medium was developed in this way for conifer species such as *A. lasiocarpa* (Kvaalen et al., 2005) and *Pinus oocarpa* (Lara-Chavez et al., 2011). However, for both species, the novel medium was ineffective for embryogenic tissue initiation, showing only potential for enhanced tissue proliferation and somatic embryo maturation.

### Proliferation of Embryogenic Tissue

Culture medium DCR (Gupta and Durzan, 1985) containing 2,4-D (9 μM) and BA (2.2 μM) used for initiation of somatic embryogenesis has also been found suitable for the maintenance of embryogenic tissue in *P. nigra* (Salajová et al., 1999; Salajová and Salaj, 2005). Subcultivation of tissue at regular intervals every 2–3 weeks resulted in vigorous growth and appearance of typical conifer embryogenic tissue, white and mucilaginous (Figure 1B). Transfer of tissue to fresh medium could be delayed by a few days but no longer than 4 weeks. Prolonged cultivation on solid medium (more than 4 weeks) caused browning and degeneration of the tissue. The tissue could be maintained by regular subcultivation for 2 or 3 years but the culture growth and tissue survival during this period were cell line dependent.

Microscopic examination of proliferating tissue revealed variable micromorphology of somatic embryos depending on cell lines. The cell lines were categorized based on the micromorphology of stage 1 somatic embryos (von Arnold and Hakman, 1988) (Salajová and Salaj, 2005). Group 1 cell lines contained well-formed bipolar early somatic embryos composed of an embryonal part with a regular outline with attached long vacuolated suspensor cells arranged into bundles (Figure 1C). Group 2 cell lines were characterized by the presence of less organized somatic embryos. The embryonal part consisted of a loosely aggregated mass of meristematic cells and the attached suspensor cells lacked organization into bundles (Figure 1D). In cell lines categorized as group 3, mostly unorganized meristematic cell aggregates were observed. Bipolar structures, consisting of few meristematic cells connected to 1–2 long vacuolated cells, were also present. The somatic embryos differed in micromorphology among cell lines, but within each cell line they were similar. The micromorphology of somatic embryos affected the maturation capacity of cell lines (Salajová and Salaj, 2005). Only cell lines with somatic embryos categorized as group 1 produced cotyledonary somatic embryos capable of germination and somatic seedling regeneration. As already mentioned, some cell lines can be maintained for a longer period (up to 2 years) but longer cultivation results in a decrease or complete loss of maturation capacity and disintegration of bipolar organization (Klubicová et al., 2017).

Suspension cultures of embryogenic tissue were established by resuspending defined amounts of tissue (0.5, 1.0, and 2.5 g) in 25 ml of liquid DCR medium of the same composition used for growth on solid medium (Salaj et al., 2007a). These cultures were cultured on a rotary shaker at 100–110 rpm in Erlenmeyer flasks at 25°C in the dark. For growth evaluation the settled cell volume (SCV) as a non-destructive quantitative parameter was used. The initial “inoculum” weight affected the culture growth in liquid medium and 0.5 g of tissue as “inoculum” was insufficient for continuous proliferation in most of the cell lines. Higher initial weight of “inoculum” (1 or 2.5 g) resulted in better growth, obtaining higher SCV values, but still profound differences were observed among cell lines. The structure of early somatic embryos showed similar features as on solidified media. Somatic embryo maturation in suspension cultures was limited and could not be completed. The early somatic embryos present in suspension cultures developed only to the cotyledonary stage and were able to regenerate plantlets only when transferred to solid media.

### Somatic Embryo Maturation and Germination

Somatic embryo maturation in pine species is stimulated by transfer of proliferating tissue to medium devoid of auxins and cytokinins and supplemented by abscisic acid (ABA). The optimum concentration of ABA varies depending of species (Aronen et al., 2009; Montalbán et al., 2010) and may differ between two cell lines of the same species (Carneros et al., 2017).

Another important factor involved in somatic embryo maturation in pines is the water availability. In *P. strobus*, media with a high gellan gum concentration (1%) promoted the maturation of large numbers of somatic embryos in four tested cell lines (Klimaszewska and Smith, 1997). The increased medium gel strength caused lowered water content available to the cells (Klimaszewska et al., 2016). Somatic embryo maturation is genotype dependent and considerable variations in cotyledonary somatic embryo yield were noticed among *Pinus* species, cell lines and maturation treatments. The best maturation treatments yields - number of cotyledonary somatic embryos calculated per 1 g of fresh mass recorded for several species are: 187 for *P. pinaster* (Alvarez et al., 2013), 127 for *P. sylvestris* (Aronen et al., 2009), 1550 for *P. radiata* (Montalbán et al., 2010), 384 for *P. pinea* (Carneros et al., 2017), 287 for *P. luchuensis* (Hosoi and Maruyama, 2012).

Cotyledonary somatic embryos in pine closely resemble their zygotic counterparts in respect to structural organization and biochemical characteristics. Zygotic and somatic embryos of *P. pinaster* exhibited similar carbohydrate and protein contents. The high level of similarity was also supported by proteome profiling (Morel et al., 2014b).

For germination, individual cotyledonary somatic embryos are cultured on medium containing no plant growth regulators. The germination can be improved by post maturation treatments. For many pine species desiccation applied to somatic embryos significantly improved germination. Slow desiccation at high relative humidity increased the germination percentages from 21 to 71% in Japanese pines. Contrary, fast desiccation was lethal, causing mortality of somatic embryos (Maruyama and Hosoi, 2012).

**FIGURE 2:**
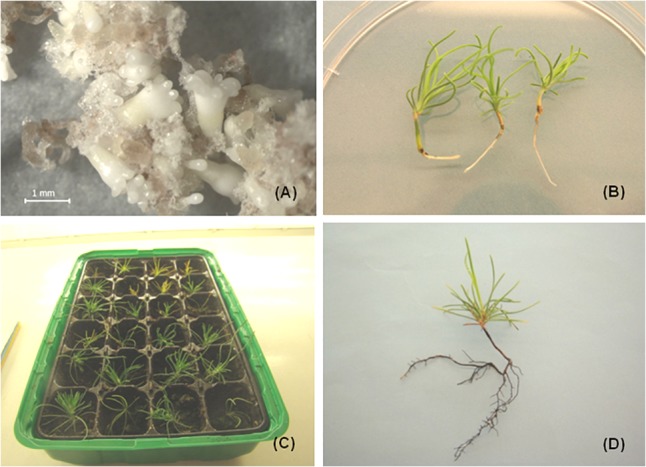
Somatic seedlings development in *P. nigra*. **(A)** Cotyledonary somatic embryos developed on maturation medium (after 8 weeks of culture), **(B)** somatic seedlings with green cotyledons and radicula, **(C)** somatic seedlings transferred to soil, **(D)** somatic seedling after growing in soil for approximately 3–4 months.

Somatic embryo development (in *P. nigra*) occurred after transfer of tissue from proliferation medium to maturation medium and the developmental stages were identified according to von Arnold and Hakman (1988). In earlier experiments, the maturation medium was supplemented with low concentrations of the PGRs abscisic acid (ABA, 0.38 to 38 μM), BA (0.44 μM), and kinetin (0.43 μM). The development of somatic embryos was limited, reaching mostly stage 2 and no plantlets (somatic seedlings) were obtained. Supplementation of the maturation medium with a higher ABA concentration (95 μM) and maltose (3, 6, and 9%) led to the formation of well-developed cotyledonary somatic embryos capable of regenerating somatic seedlings. The somatic embryo development was cell line dependent and was affected by maltose concentration in the maturation medium and the desiccation treatment (Salajová et al., 1999). The somatic embryo maturation was also affected by structural aspects of early somatic embryos present in proliferating tissue (Salajová and Salaj, 2005). The mentioned approaches required a post maturation step with the culture of developing somatic embryos on media lacking ABA and a lowered concentration of maltose (Salajová et al., 1999; Salajová and Salaj, 2005). In further experiments the post-maturation step was removed and the maturation medium was supplemented with a higher concentration of ABA (95 μM) and 1% gellan gum (Gelrite^TM^, Duchefa). In this case the cotyledonary embryos (Figure 2A) were transferred from maturation medium directly to germination medium. This approach also resulted in somatic seedling regeneration (Salaj et al., 2014). Using any of the mentioned approaches, the maturation was cell line dependent and plantlets were only obtained for some cell lines. Moreover, somatic embryo development was asynchronous.

For germination, well developed somatic embryos possessing at least four cotyledons were selected. The germination occurred in the dark for 7–9 days followed by cultivation in the light (16-h photoperiod). The germination frequencies ranged from 30.7 to 71.3% for DCR medium and from 24.5 to 60.3% for MLV. The germination of somatic embryos on hormone free media resulted in somatic seedling regeneration (Figure 2B). The regenerated somatic seedlings were transferred to soil (Figure 2C). After approximately 4–6 months of growth in soil well developed root systems were observed (Figure 2D).

### Cryopreservation of Embryogenic Tissue

In recent two decades optimized cryopreservation protocols were elaborated for many plant species, including conifers. Cryopreservation used for conifer embryogenic tissue is based on the slow-freezing method (Häggman et al., 1998; Fraga et al., 2016b; Nunes et al., 2017). Several pine species were cryopreserved for different periods of time with relatively high recovery frequencies reaching values 87% for *P. pinea* (Carneros et al., 2017), 100% for *P. pinaster* (Alvarez et al., 2012) and 100% for *P. radiata* (Lineros et al., 2018). In most species the maturation ability of cryopreserved and recovered tissues was restored.

**FIGURE 3:**
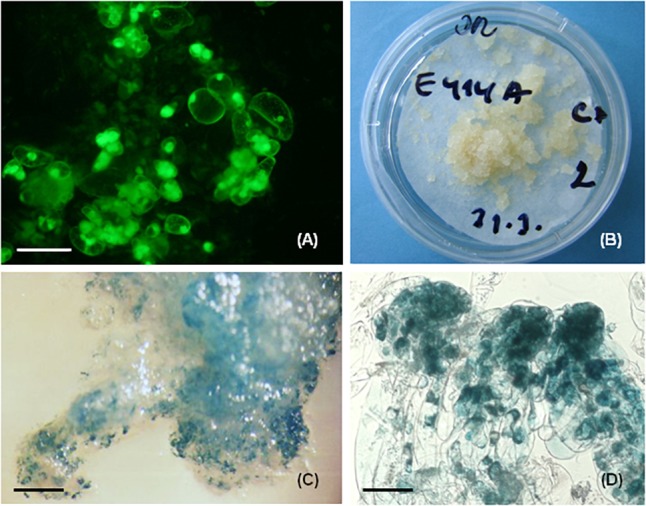
Cryopreservation and genetic transformation in *P. nigra*. **(A)** FDA staining immediately after cryopreservation, the fluorescence signal was concentrated in the meristematic embryonal cells that survived cryopreservation, **(B)** regrowth of embryogenic tissues after 3 weeks of thawing, **(C)** GUS expression in recovered transgenic embryogenic tissue, **(D)** GUS expression in early somatic embryos, the GUS activity was concentrated mostly in embryonic cells. Scale bars: **(A)** = 50 μm, **(C)** = 800 μm, **(D)** = 100 μm.

For *P. nigra* the method included preculture of well growing tissue on proliferation medium containing 180 g.l^-1^ sucrose and 7.5% DMSO for up to 1 h (Salaj et al., 2011). Pretreatment using maltose and sorbitol incorporated into the solid medium was also tested for some cell lines. The duration of storage in liquid nitrogen lasted from 30 min to 1 year. Investigation immediately after thawing showed disintegration of somatic embryo structure. The fluorescence signal was apparent mostly in meristematic embryonal cells, and the long vacuolated suspensor cells were disrupted (Figure 3A). Tissue regeneration began soon after thawing, around 3–4 days, and massive growth was observed after 2 weeks of cultivation (Figure 3B). 56 cell lines were cryopreserved and regrowth was observed in 45 of them (80.36%). The tissue regrowth was cell line dependent with individual frequencies of 100% (15 cell lines), 80–92% (11 cell lines) 60–78% (8 cell lines), and less than 60% (11 cell lines). Tissue regrowth was also affected by pretreatment, with sorbitol giving lower regrowth frequencies compared with maltose or sucrose. The growth of regenerated tissue was evaluated as fresh and dry mass increase in the post thaw period (3 months after thawing) and compared to the growth of non-pretreated and non-cryopreserved tissue (control 2). No statistically significant differences in growth were observed between cryopreserved and regrown tissue and control 2, suggesting the cryopreservation did not negatively affect the proliferation. Similarly, during the post-thaw period the structure of somatic embryos was recovered and somatic embryos showing similar organization prior to cryopreservation were observed in regrown tissue. The cryopreserved tissue was capable of maturation and somatic seedling development (Salaj et al., 2007b). No correlation was found between maturation capacity of cell lines and their cryotolerance (Salaj et al., 2012).

### Genetic Transformation

Apart from being an efficient plant regeneration system, somatic embryogenesis is also an enabling tool for genetic transformation studies. Embryogenic tissue of pine species was involved into genetic transformation studies using biolistic technique or *Agrobacterium*-mediated transformation. Biolistic transformation resulted in transient expression of *uid*A reporter gene in *P. radiata* (Walter et al., 1994) and in *P. sylvestris* (Häggman and Aronen, 1998). Using the same technique, stable transformation and transgenic somatic seedling regeneration was reported for *P. radiata* (Walter et al., 1998). Successful *Agrobacterium tumefaciens*-mediated transformation has been achieved in *P. radiata* (Walter et al., 1999; Cerda et al., 2002), *P. strobus* (Levee et al., 1999), and *P. pinaster* (Alvarez and Ordas, 2013).

For *P. nigra* the biolistic method was tested and two embryogenic cell lines, E103 and E104, were involved as targets in the study (Salaj et al., 2005). The plasmid pCW 122 (Walter et al., 1994) bearing the reporter *uid*A gene encoding β-glucuronidase under the control of double CaMV 35S promoter and *npt*II gene controlled by single CaMV 35S promoter was used. For bombardment, the biolistic particle delivery system (PDS 1000/HE System, Bio-Rad, Hercules, CA, United States) was used. Tissue recovery occurred on selection medium containing geneticin (25 mg.l^-1^). 10–12 weeks after bombardment only cell line E104 showed regeneration. Histochemical assessment revealed 434 to 588 GUS expressing spots in 1g of investigated tissue (Figure 3C). The intense blue staining was visible mostly in the meristematic embryonal part and weak or no staining was present in suspensor cells (Figure 3D). PCR analyses confirmed the presence of *uid*A as well as *npt*II genes in regenerated geneticin-resistant tissue.

### Proteomic Analysis

To gain deeper insight into somatic embryogenesis in conifers, a proteomic approach has been used in *Picea glauca* (Lippert et al., 2005), *Picea asperata* (Jing et al., 2017), *Larix* x *eurolepis* (Teyssier et al., 2014), *P. pinaster* Ait. (Morel et al., 2014a) and *Araucaria angustifolia* (dos Santos et al., 2016; Fraga et al., 2016a). Proteomic studies in *P. pinaster* Ait. were focused on the comparison of somatic and zygotic embryos in cotyledonary developmental stage (Morel et al., 2014b) or on the characterization of molecular mechanisms role in the development and maturation of somatic embryos (Morel et al., 2014a). Proteome profiles showed high level of similarity in cotyledonary zygotic and somatic embryos (Morel et al., 2014b). In detailed study of Morel et al. (2014a), somatic embryos of *P. pinaster* matured in different conditions of water availability. The maturation medium was gelified with gellan gum 4 g.l^-1^ (indicated as 4G) or 9 g.l^-1^ (indicated as 9G). In 4G conditions the somatic embryos developed abnormally, showing dead cells in meristematic centers as well as in suspensor. In 9G conditions the somatic embryos developed as well organized structures with enlarged meristematic center and vacuolized suspensor cells arranged into bundles. Comparative proteomic study detected differences for 83 spots, 35 with greater abundance on 4G and 48 on 9G. Protein identification was successful for 56 spots. The results indicate enhanced glycolysis under unfavorable conditions of maturation (medium G4), leading to proliferation. On the basis of the study, the germin-like protein and ubiquitin protein ligase were proposed as potential marker genes of effective somatic embryo development in favorable conditions (Morel et al., 2014a).

To determine proteins related to embryogenic capacity in *P. nigra*, a proteomic approach based on 2-dimensional electrophoresis was applied (Klubicová et al., 2017). Two cell lines (E362 and E366) with high embryogenic capacity were selected to eliminate the influence of genotype/cell line. Three different tissues – embryogenic tissue with high embryogenic capacity, non-embryogenic tissue and tissue after the loss of embryogenic capacity – from both cell lines were analyzed (Klubicová et al., 2017). Investigated tissue showed distinct structural features. 108 and 110 protein spots were differentially accumulated in E362 and E366 cell lines, respectively, when comparing embryogenic and non-embryogenic tissue. Only 24 of them were altered in both cell lines. Many of the identified proteins were active in disease and defense mechanisms, energy metabolism and biosynthesis of cell wall components. Three protein spots were detected only in the embryogenic tissue of both cell lines. Two of them, similar to water deficit inducible protein LP3, were previously suggested as playing a role in cell wall expansion during crown wood tissue formation (Paiva et al., 2008). The third was identified as a protein which contains the 3-demethylubiquinone-9 3-methyltransferase domain and is involved in ubiquinone biosynthesis. The loss of maturation capacity was accompanied by changes in 35 and 38 protein spots in E362 and E366 cell lines, respectively. Only two of them were altered in both cell lines, suggesting a non-uniform process of aging. The impact of cell line on embryogenic capacity was manifested, despite similarly high embryogenic capacity in both analyzed cell lines.

## Somatic Embryogenesis in the Hybrids *Abies alba* x *A. cephalonica* and *Abies alba* x *A. numidica*

### Initiation and Proliferation of Embryogenic Tissue

In the genus *Abies* somatic embryogenesis has been initiated in several species, including *A. alba* (Schuller et al., 1989; Zoglauer and Reuther, 1996; Nawrot-Chorabik, 2008; Vooková and Kormutak, 2009), *A. balsamea* (Guevin et al., 1994), *A. cephalonica* (Krajňáková et al., 2008), *A. fraseri* (Guevin and Kirby, 1997; Pullmann et al., 2016), *A. lasiocarpa* (Kvaalen et al., 2005), *A. nordmanniana* (Nörgaard and Krogstrup, 1991; Misson et al., 2006; Nawrot-Chorabik, 2016), *A. numidica* (Vooková et al., 2001, Vooková et al., 2003), *A. cilicica* (Vooková and Kormut’ák, 2003), and hybrid firs (Gajdošová et al., 1995; 1997/1998; Korecký and Vitámvás, 2011). It is worthy of mention that in *A. alba* somatic embryogenesis was initiated as early as 1986 (Erdelský and Barančok, 1986). The authors described initiation of “white special tissue” but did not recognize the embryogenic nature of this tissue. For most of the species belonging to genus *Abies* or *Abies* hybrids, cytokinin alone in the nutrient medium was sufficient for embryogenic tissue initiation, although differences were noticed between the effects of different types of cytokinins. In *A. nordmanniana* TDZ (0.1 μM) and BA (5 μM) were equally effective (Nörgaard and Krogstrup, 1991), in *A. balsamea* 2-iP gave higher initiation frequencies in comparison to BA or TDZ (Guevin et al., 1994). Auxin in the culture medium as the sole PGR or in combination with cytokinins was not necessary for embryogenic culture initiation or proliferation and its incorporation into the culture medium resulted in tissue growth cessation in *A. nordmanniana*. This phenomenon is explained by an optimal self-sustained auxin production (Nörgaard and Krogstrup, 1991).

The auxin demand of tissue could be a species-dependent phenomenon, as proliferation of embryogenic cell lines of *A. balsamea* occurred on media containing only BA (10 μM ) as well as on medium containing 4.5 μM BA combined with 10 μM NAA (Guevin et al., 1994). Similarly, Vondráková et al. (2011) demonstrated the positive role of 2,4-D for embryogenic tissue proliferation in *A. alba*, although the concentration used was very low (0.25 μM 2,4-D combined with 2 μM BA and 2 μM kinetin).

In our work with somatic embryogenesis in *Abies* hybrids, hybrid seeds were obtained from female cones of *A. alba* Mill. pollinated with *A. cephalonica* Lond. or *A. numidica* DeLann. For embryogenic tissue initiation juvenile explants as megagametophyte with developing zygotic embryos, mature zygotic embryos excised from seeds and cotyledon segments dissected from seedlings or emblings (somatic seedlings) were used.

#### Immature Zygotic Embryos

The immature zygotic embryos were enclosed in megagametophytes isolated from seeds excised from young cones. Different basal media compositions with different PGR concentrations were tested, including DCR (Gupta and Durzan, 1985), SH (Schenk and Hildebrandt, 1972), and 1/2 LM (Litvay et al., 1981). Best initiation results were obtained when using SH medium supplemented with BA (4.4 μM) with frequencies ranging from 4.4 to 38.1% (*A. alba* x *A. cephalonica*) and 15.8–44.6% (*A. alba* x *A. numidica*). Very limited initiation was obtained using DCR and no initiation was recorded on 1/2 LM (Salajová et al., 1996).

#### Mature Zygotic Embryos

Hybrid seeds (*A. alba* x *A. cephalonica*) used in experiments were stored for 6 months to 4 years and after surface sterilization the mature embryos were excised and cultured on SH medium (Schenk and Hildebrandt, 1972) containing BA (4.4 μM) as the sole plant growth regulator. Embryos dissected from seeds stored for 6 months or 1 year produced embryogenic tissue in frequencies ranging from 27.2 to 29%. Prolonged storage of seeds (4 years) resulted in loss of explant competence to produce embryogenic tissue. The somatic embryos differentiated on the hypocotyl of isolated embryos as single structures and in contact with nutrient medium they proliferated and gradually the explants were overgrown with embryogenic tissue (Salaj and Salaj, 2003).

#### Cotyledons Dissected From Seedlings or Somatic Seedlings

The zygotic seedlings were obtained as a result of *in vitro* germination of hybrid seeds (*A. alba* x *A. cephalonica*). The somatic seedlings developed from somatic embryos of cell lines AC1, AC2, AC5, AC78, AC79 (*A. alba* x *A. cephalonica*) and AN72 (*A. alba* x *A. numidica*). Cotyledons dissected from mentioned plantlets were cultivated on DCR medium (Gupta and Durzan, 1985) supplemented with BA (4.4 μM). The initiation frequency on cotyledons dissected from seedlings of zygotic origin was very low. Out of 103 cultured explants only two formed embryogenic tissue (1.94%) and only one of them was maintained as a cell line (AC13). The embryogenic potential of cotyledons of somatic seedling origin was higher and out of six genotypes tested five yielded embryogenic tissue with initiation frequencies ranging from 1.24% (AC1) to 24.28% (AC78) (Salajová and Salaj, 2001). The embryogenic structures appeared on the surface of cotyledons after 2–5 months of cultivation. Histological studies revealed intensive cell division activity in epidermal and subepidermal layers of the cotyledons. As a result of such activity, meristematic cell clusters formed and subsequently nodular structures developed, emerging from the epidermis. These structures differentiated firstly into polarized structures, with clearly distinguishable vacuolated suspensor-like cells and tightly packed meristematic cells in the embryonic part. Their further development resulted in typical somatic embryo differentiation.

#### Proliferation of Embryogenic Tissue

**FIGURE 4:**
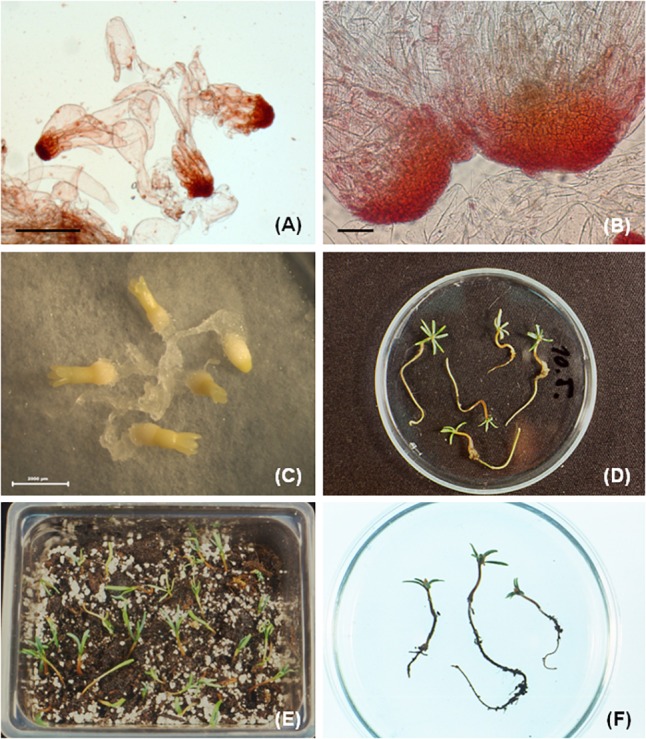
Somatic embryogenesis in *Abies* hybrids. **(A)** Bipolar somatic embryos observed in cell line AN72, **(B)** besides bipolar structures also huge embryonal complexes are typical for hybrid *Abies* embryogenic tissues, **(C)** cotyledonary somatic embryos after 8–9 weeks of maturation, **(D)** regenerated somatic seedlings after germination, **(E)** somatic seedlings transferred to soil, **(F)** somatic seedlings growing in soil for 3–4 months (**F**, *with permission from Salaj et al. Acta Biol. Cracov., Ser. Bot. 46, 159-167, 2004*). Scale bars: **(A)** = 1000 μm, **(B)** = 200 μm, **(C)** = 2000 μm.

The initiated tissue was separated from primary explants and cultured as individual cell line. For proliferation and long-term maintenance, SH medium supplemented with BA (4.4 μM) as the sole PGR was used. The tissue shared similar features, irrespective of their origin (immature or mature zygotic embryos or cotyledons). The white embryogenic tissue was rapidly growing and required transfer to fresh media after 3 weeks of cultivation. The survival of tissue during maintenance was cell line dependent. Some tissue died after 5–6 months of culture, others proliferated for several years. In the proliferating tissue of hybrid firs, besides the typical bipolar structures (Figure 4A), huge polyembryonal complexes were often observable. In these structures the meristematic “heads” were joined creating a broad cell “package” connected to long vacuolated suspensor cells (Figure 4B). Structural investigation revealed the embryonal part is composed of meristematic cells. Isodiametric, highly cytoplasmatic cells with a prominent, centrally located nucleus and rich in cell structures such as proplastids, mitochondria, dictyosomes, endoplasmic reticulum, ribosomes and small vacuoles were observed. The cell wall was thin and contained numerous plasmodesmata. The elongated suspensor cells were highly vacuolated with a thin layer of cytoplasm in the cell periphery (Jásik et al., 1999).

### Maturation and Germination of Somatic Embryos

Successful somatic embryo development into cotyledonary stage, terminated by somatic seedlings regeneration in *Abies*, has been reported for *A. alba* (Hristoforoglu et al., 1995; Krajňáková et al., 2013), *A. nordmanniana* (Nörgaard, 1997), *A. fraseri* (Guevin and Kirby, 1997; Pullmann et al., 2016).

Abscisic acid is an important requirement for somatic embryo maturation in the genus *Abies*. In early work on *A. balsamea*, Guevin et al. (1994) indicated factors in addition to ABA may affect somatic embryo development. The importance of carbohydrate source was emphasized in *A. nordmanniana* (Nörgaard, 1997). Maltose, irrespective of concentration, was superior to sucrose in terms of the number of mature somatic embryos obtained. Contrary, higher germination frequencies were obtained on media containing sucrose (40% versus 26% for maltose). For this species, the promoting effect of PEG-4000 as a non-penetrating osmoticum was also apparent, mostly when PEG-4000 was applied during the first 2–6 weeks of maturation. Over-proliferation of embryogenic tissue is an accompanying phenomenon during maturation of somatic embryos in *Abies*. Reduction of over-proliferation was a further positive effect of PEG (Nörgaard, 1997).

In *A. cephalonica* the beneficial effect of maltose over sucrose was apparent during the maturation period. Embryogenic tissue growing on maltose-containing medium yielded more mature somatic embryos than those growing on sucrose, although their germination frequencies were significantly lower than those matured in the presence of sucrose (Krajňáková et al., 2008). In the mentioned species the somatic embryo maturation was cell line-dependent, suggesting the important role of genotype.

Nörgaard and Krogstrup (1995) mentioned that one problem of somatic embryo development in *A. nordmanniana* is the high frequency of aberrant somatic embryos. Based on morphological observation and environmental scanning electron microscopic studies, Vlasinova et al. (2017) found the most frequent abnormalities in somatic embryo development were cup-shaped cotyledons, fused cotyledons, single cotyledon, meristem-less somatic embryos and disrupted meristems. For *Abies* hybrids, mostly fused cotyledons as well as the meristem-less abnormal structures were frequently observed. The abnormal structures exhibited destruction of root meristem and absence of apical meristem (Jásik et al., 1999).

Embryogenic tissue of hybrid fir, regardless of origin, from immature as well as mature zygotic embryos or cotyledons, produced somatic embryos in different developmental stages. Precotyledonary somatic embryos developed in all tested cell lines (in relatively high numbers) and their further development into cotyledonary stage was restricted. In cell lines initiated from mature zygotic embryos using medium supplemented with ABA (38 μM) 7.5% PEG-4000 and 3% sucrose, 27–67% of precotyledonary somatic embryos reached the cotyledonary developmental stage depending on the cell line. Somatic embryos that did not reach the cotyledonary developmental stage showed abnormal features (Salaj and Salaj, 2003). The role of carbohydrates in somatic embryo development of hybrid firs has also been tested (Salaj et al., 2004). Sucrose, maltose and glucose in three different concentrations (3, 6, and 9%) were used. These maturation treatments resulted in precotyledonary somatic embryo development. At the lower carbohydrate concentration (3%), cotyledonary somatic embryos developed sporadically, and higher concentrations of carbohydrates yielded only degenerated, abnormal somatic embryos. Differentiation of fully developed cotyledonary embryos (Figure 4C) was achieved by incorporation of polyethylene glycol (PEG-4000 at 0, 5, 7.5, and 10%) combined with the carbohydrates glucose, sucrose and maltose (at 3%) in the maturation medium. PEG-4000 and carbohydrate stimulated somatic embryo maturation in a concentration dependent manner. Higher concentrations of PEG-4000 (7.5 and 10%) combined with 3% maltose yielded 144 and 156 cotyledonary somatic embryos, respectively, calculated per Petri plate containing 1.2 g of inoculum. Histological observations revealed the regularly shaped cotyledonary somatic embryos were characterized by an internal organization similar to that of their zygotic counterparts. The shoot apical meristem was surrounded by a ring of cotyledons, in the root pole, root meristem, root cap, and columella were present (Salaj et al., 2004).

After the 3 weeks desiccation treatment in the dark, somatic embryos were transferred to germination medium (DCR) devoid of ABA and PEG-4000 (Salaj et al., 2004). The germination medium contained carbohydrates glucose, sucrose, maltose (each at 3%) and was supplemented with 1% activated charcoal. The germination of somatic embryos was influenced by the maturation treatment. Relatively high germination frequencies (47.61–78.92%) were obtained for somatic embryos matured in the presence of 7.5 or 10% PEG-4000. The 1st week of germination occurred in the dark and after that the embryos were transferred to light. At day 21 of germination, most of the germinating embryos had 0.5–1 cm long radicles and green cotyledons (Figure 4D). In this developmental stage the germinating plantlets were transferred from Petri plates to Magenta baby food jars containing medium of the same composition as used for germination. The transfer resulted in transient decline of growth, lasting approximately 3 weeks, after which plantlet elongation was observed. Eventually, the regenerated somatic seedlings were transferred to soil (Figure 4E,F).

### Cryopreservation of Hybrid Firs Embryogenic Tissue

Cryopreservation is safe and cost-efficient technique for long-term storage of plant genetic resources. The technique was successfully applied to several species of *Abies* as *A. alba* (Krajňáková et al., 2013), *A. cephalonica* (Aronen et al., 1999; Krajňáková et al., 2011), *A. nordmanniana* (Nörgaard et al., 1993; Misson et al., 2006), *A. fraseri* (Pullmann et al., 2016). The advantage of embryogenic tissue cryopreservation is that somatic embryos in very early developmental stage (proliferation) are cryopreserved. Another advantage is that the tissues can be stored in liquid nitrogen until field tests prove the best cell lines, producing somatic seedlings capable of growth in field conditions. After thawing, the tissue regeneration is relatively rapid and the tissues can be maintained on proliferation medium, regularly used for their *in vitro* maintenance. For *Abies* embryogenic tissues, the slow-freezing method was mostly used. The regrowth of tissues after thawing is strongly genotype-dependent. In *A. fraseri*, out of 22 cryopreserved cell lines, 12 survived cryopreservation (Pullmann et al., 2016). In *A. alba* 12 cell lines were cryopreserved for 6 years and 4 of them recovered after thawing. Cotyledonary somatic embryos were produced in 2 recovered cell lines (Krajňáková et al., 2013).

An important point of cryopreservation is the genetic fidelity of plant material recovered from liquid nitrogen. In *A. cephalonica* genetic variation performed by RAPD assay was detected in control samples (pretreated but not frozen), and no variation was detected in retrieved tissues (Aronen et al., 1999). At a later stage, in the same species genetic variability was detected in one of regenerated cell line cryopreserved for 6 years. A possible reason could be the cryopreservation or somaclonal variation during proliferation (Krajňáková et al., 2011).

In cryopreservation experiments focused on hybrid firs, four cell lines: AC78, AC1, AC4 (hybrid *A. alba* x *A. cephalonica*) and AN72 (hybrid *A. alba* x *A. numidica*) were included, using the slow-freezing method (Salaj et al., 2010). The tested cell lines withstood cryopreservation and the regrowth frequencies were dependent on cell line as well as treatment. Cell lines AN72 and AC78 showed relatively high cryotolerance (regrowth 83–100%), cell lines AC1 and AC4 were more sensitive to cryopreservation (regrowth frequencies 37.5–100%). Growth analysis in regenerated tissue showed that in most cases cryopreservation had no negative effect on tissue growth in the post-thaw period. The cryopreserved tissue developed mature somatic embryos capable of somatic seedling regeneration. RAPD analysis did not reveal any changes in genetic fidelity of cryopreserved tissue (Salaj et al., 2010). Microscopic observation of cryopreserved tissue showed disintegration of early bipolar structures immediately after cryopreservation. In the regrowth period following removal from cryopreservation, the disintegrated structures regenerated and regained the original bipolarity (Salaj et al., 2016).

### Genetic Transformation

During the last two decades, progress has been achieved in genetic transformation studies of conifer species, including representatives of the genus *Abies*. Find et al. (2005) reported successful genetic transformation of *A. nordmanniana* using the biolistic method and Lee et al. (2014) obtained transformed embryogenic tissue of *A. koreana* Wil. using *Agrobacterium tumefaciens* mediated transformation. In *A. nordmanniana*, the embryogenic cultures, as well as plantlets regenerated from such tissue, expressed the transgenes even 5 years after transformation (Find et al., 2005). A prerequisite for successful genetic transformation is using plant material with high regeneration ability. Rapidly growing embryogenic tissue producing early somatic embryos with the ability to regenerate somatic seedlings represents such material (Lee et al., 2014). The success of transformation in *A. koreana* Wil. was also dependent on the *Agrobacterium* strain. A total of 48 putative transgenic embryogenic cell lines were selected. The variable transgene expression, detected among cell lines, could be explained by the number of copies integrated into the genome (Klimaszewska et al., 2003; Lachance et al., 2007).

The embryogenic tissue of cell lines AC1, AC78 (hybrid *A. alba* x *A. cephalonica*) and AN72 (hybrid *A. alba* x *A. numidica*) were characterized by high regeneration potential and were capable of producing somatic seedlings. Cocultivation with *Agrobacterium tumefaciens* (strain AGL0) containing plasmid pTS2, a derivative of the binary vector pBinPlus, was used in our investigation. It contained the neomycin phosphotransferase II (*nptII*) gene under the control of *nos* promoter and intron-containing β-glucuronidase (*gus*) reporter gene under the control of double CaMV 35S promoter. After cocultivation the tissue was washed in distilled water, then in a tetracycline solution (25 mg.l^-1^) and again in distilled water. Selection occurred on DCR proliferation medium containing geneticin (10 mg.l^-1^).

Regeneration of tissue started after 1 week of culture on selection medium and growth was relatively fast. The transgenic nature of regenerated tissue was proved by evaluation of GUS activity. The intensity of GUS staining was concentrated mainly in the embryonal “head” of early somatic embryos. Cotyledonary somatic embryos developed on maturation medium containing ABA and PEG-4000 and somatic seedling regeneration also occurred. The presence of both *nptII* and *gus* was confirmed by PCR analyses (but these genes were present only in 11 out of 36 analyzed plantlets), suggesting the embryogenic sublines were chimeras and the origin of emblings can be traced in transformed as well as non-transformed cells (Salaj et al., 2009).

## Conclusion

Conifers are classified as recalcitrant species for vegetative propagation through *in vitro* culture. The enormous effort devoted to micropropagation *via* somatic embryogenesis in recent years has resulted in the development of functional protocols for a number of conifer species. Moreover, progress has also been achieved in the application of biotechnological tools such as genetic transformation and cryopreservation. Somatic embryogenesis is a special route of *in vitro* plant regeneration applicable for basic physiological and biochemical studies and structural aspects of early plant development. The application of modern biotechnological methods may play a crucial role in alleviating the negative effect of expected climate change and the human induced environmental problems (Fenning, 2012). For the conifers *P. nigra* and *Abies* hybrids, protocols focused on somatic embryogenesis initiation as well as somatic seedling development were developed. For cryopreservation of embryogenic tissue, the slow-freezing technique was successfully applied with recovery frequencies 80.36–100%. The gene transfer experiments by particle bombardment or *Agrobacterium*-mediated resulted in stable transformation of the reporter *gus* gene in embryogenic tissue. Although somatic seedling regeneration has been achieved in pine as well as *Abies* hybrids, some unresolved problems such as low initiation frequencies in pine, limited maturation ability and less successful transfer to soil, still remain. These phenomena are limiting factors for complete somatic seedling regeneration and implementation of somatic embryogenesis for mass propagation. To overcome these disadvantages, further studies focused on physiological, as well as structural aspects of somatic embryogenesis, are necessary. Recently proteomic studies were also undertaken to gain a deeper insight into the process of somatic embryogenesis in pine species.

## Author Contributions

TS outlined the paper area and prepared preliminary manuscript text. KK, RM, and JS contributed actively to the proposed manuscript text, and designed tables and figures.

## Conflict of Interest Statement

The authors declare that the research was conducted in the absence of any commercial or financial relationships that could be construed as a potential conflict of interest.
